# Effect of Laser Pulse Width on Cutting Quality and Efficiency in CFRP: Mechanism and Optimization

**DOI:** 10.3390/ma18204707

**Published:** 2025-10-14

**Authors:** Chunmeng Chen, Long Chen, Guojun Zhang, Yu Huang, Huijuan Ma, Youmin Rong

**Affiliations:** 1 School of Mechanical Science and Engineering, Huazhong University of Science and Technology, Wuhan 430074, China; chenchunmeng@126.com (C.C.); longchen_hust@163.com (L.C.); zhanggj.hust@gmail.com (G.Z.); yuhuang7208@163.com (Y.H.); 2 State Key Laboratory of Intelligent Manufacturing Equipment and Technology, Huazhong University of Science and Technology, Wuhan 430074, China; 3 Guangdong Provincial Key Laboratory of Manufacturing Equipment Digitization, Guangdong HUST Industrial Technology Research Institute, Dongguan 523808, China; 4 School of Automotive Engineering, Wuhan University of Technology, Wuhan 430070, China; mahuijuan21@whut.edu.cn

**Keywords:** laser cutting, CFRP, pulse width, heat-affected zone

## Abstract

This study systematically investigates the influence of laser pulse duration on cutting efficiency, heat-affected-zone (HAZ) formation, and mechanical integrity during carbon fiber-reinforced polymer (CFRP) laser cutting. Three distinct pulse-width lasers—picosecond, nanosecond, and quasi-continuous-wave (QCW)—are compared. Results show that pulse duration governs material removal mechanisms and HAZ extent: the nanosecond laser achieves the smallest HAZ and minimal porosity; the picosecond laser exhibits limited thermal accumulation due to low average power; and the QCW laser induces the largest HAZ (11.6 times that of the nanosecond laser) and significant porosity. Cutting efficiency scales inversely with pulse width, with single-hole processing times of 480.4 s for picosecond-laser cutting, 76.8 s for nanosecond-laser cutting, and 4.028 s for QCW-laser cutting, reflecting a transition from thermal ablation to mechanical spallation. Mechanical testing reveals that while tensile and flexural strengths vary by less than 5% across laser types, damage morphology and failure modes differ significantly. In situ digital image correlation (DIC) and 3D CT imaging show that longitudinal plies fail via fiber pull-out, whereas transverse plies fail via interfacial debonding. QCW-laser-cut specimens exhibit more uniform strain distribution and higher damage tolerance. An optimized process parameter is proposed: nanosecond-laser cutting at 200 W and 20 kHz achieves a HAZ of less than 50 µm and a cutting time of less than 80 s, offering the best balance between efficiency and quality.

## 1. Introduction

Carbon fiber-reinforced polymer is an important structural material in several sectors, including automotive, aerospace, wind power, and other industries [1,2], due to its remarkable properties, such as high specific strength and specific modulus, heat resistance, corrosion resistance, and radiation resistance. CFRP components, such as aircraft wings and automotive panels, are joined by means of perforated connections, and the quality of hole processing significantly affects the connection strength [3]. Therefore, researchers are focusing on high-quality and high-efficiency processing methods for CFRP [4,5].

CFRP processing typically involves techniques such as mechanical drilling and laser cutting [6,7,8,9]. Yadav et al. [10] used a Hardinge VMC 600 II vertical machining center with a 4 mm diameter uncoated tungsten carbide twist drill bit to drill holes under three different feed rates, and performed quasi-static tension tests of the plain and open-hole specimens made of the cross ([0/90]_8_) and multi-directional ([45/−45/0/90]_4_) CFRP laminates. The results indicated that holes in CFRP reduced their strength and stiffness, with transverse cracking and delamination being the main causes of failure for open-hole [0/90]_8_ laminates, and interlaminar shear and transverse cracking being the main causes of failure for [45/−45/0/90]_4_ laminates [11,12]. Laser processing has become more prevalent in CFRP, particularly due to its high efficiency, ease of shape control, and absence of tool wear. Factors such as wavelength and pulse width greatly affect the efficiency and quality of this laser cutting quality [13,14]. Li et al. explored the use of continuous-wave fiber lasers to cut CFRP under different cutting parameters [15]. The results indicated that fiber lasers are highly efficient for cutting CFRP, with a minimum HAZ of 707 µm and surface roughness of 2.43 µm. They also studied the influence of machining quality on strain distribution and mechanical behavior of CFRP using the digital image correlation (DIC) technique [16]. The DIC technique revealed that the crack propagation and failure mode correlated well with the high level of strains developed around the hole. Short-pulse lasers can effectively reduce heat accumulation and improve cutting quality, with many researchers studying the use of nanosecond UV lasers for CFRP cutting. Xu et al. [17] used a 532 nm nanosecond laser with laser rotational cutting technology to cut holes on a 2 mm thick CFRP plate; the minimum HAZ, surface roughness, and hole taper achieved were 71.7 µm, 2.68 µm, and 0.64°, respectively. Li et al. proposed the dimethicone-assisted laser cutting [18] and a dimethicone-assisted interlaced scanning mode (DISM) [19] to mitigate surface damage during nanosecond UV laser hole cutting. The minimum HAZ was reduced by 85.59% with the dimethicone-assisted method, and 85.2% by DISM. In comparison to low-energy nanosecond lasers, high-energy long-pulse (LP) nanosecond lasers achieved keyhole mode cutting for CFRP materials. The formation of the keyhole-shaped trench was attributed to the laser ablation of the carbon fibers and the subsequent conduction of energy to the polymer matrix, thereby creating a small volume of molten material. This process allows for deep penetration of laser energy, thereby enhancing cutting efficiency compared with low-energy nanosecond lasers. Under LP mode, a 2.2 mm thick cross-ply CFRP panel was penetrated in six passes [20].

In contrast to long-pulse and short-pulse lasers, which possess significant thermal effects, ultrafast lasers exhibit the advantages of ultrahigh peak intensity and ultrashort-pulse duration, which are crucial in suppressing thermal diffusion during processing. Consequently, they serve as an effective machining tool for CFRP composites [21,22,23]. Ouyang et al. [23] utilized a 532 nm picosecond-laser machine with a “double rotation” cutting technique to cut 5 mm diameter circular holes in a 4 mm thick CFRP plate. No burrs or fractures were observed; machining accuracy and taper were 60 μm and 0.64°, respectively. As laser sources continue to advance, the advent of QCW fiber lasers has opened up new possibilities for low-damage processing of CFRP. These can function in both continuous and pulsed modes, with the peak power of the pulsed mode being several times that of the continuous mode [24,25,26]. Zhou et al. [25] achieved a minimum surface HAZ of 19.5 μm using a QCW fiber laser welding/drilling integrated machine to cut 1 mm thick CFRP in pulse mode through a multi-pass strategy and high cutting speed. Ye et al. utilized microsecond, nanosecond, and picosecond lasers to drill holes in 1 and 2.5 mm thick CFRP to investigate the effects of HAZ, sample thickness, and cutting-induced geometrical defects on the sample tensile strength [27]. They found that the tensile strength of laminates decreased with increasing HAZ width, and the effect of HAZ on the target tensile strength weakened with increasing laminate thickness.

In recent studies, laser cutting has been extensively explored, predominantly focusing on damage mechanisms and methods to suppress such damage. Few summative analyses have been conducted on the processing efficiency and quality of different laser cutting methods applied to the same CFRP specimens. This paper presents a comprehensive study on cutting 6 mm diameter circular holes in 2 mm thick CFRP using long-pulse, short-pulse, and ultrashort-pulse laser systems. The hole-making efficiency, HAZ size, sidewall roughness, and static mechanical properties of the processed specimens were statistically analyzed for each cutting method. The strain distribution around the holes was used to investigate the mechanism of crack formation and propagation under tensile load. The analysis of cutting efficiency and quality provides a valuable reference for selecting the most suitable processing method for CFRP cutting.

## 2. Experiment

### 2.1. Materials

**Figure 1:**
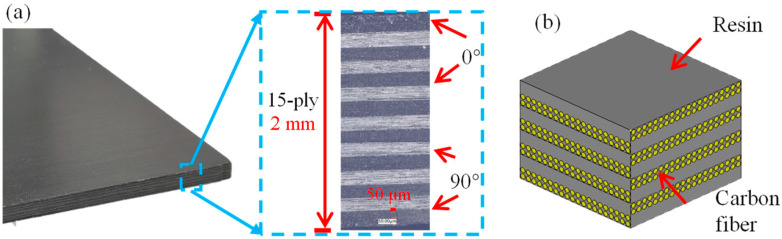
CFRP plates: (**a**) photograph; (**b**) schematic diagram.

The CFRP plates used in the three distinct pulse-width laser cutting experiments are 2 mm thick, high-performance T700 carbon fiber-reinforced epoxy-matrix composite laminates (Toray Industries, Inc., Tokyo, Japan). The material utilizes high-strength carbon fibers of 12 K tows as the reinforcement, is designed according to the interlaced layup of [0°/90°], and is prepared by a curing process involving prepreg layup and autoclave molding. This process ensures that the resin fully flows and infiltrates the fibers, achieving complete curing, thereby yielding composite materials with excellent interfacial bonding performance and low porosity. Figure 1 shows the detailed structural diagram and photographs of the carbon fiber composite laminate plate studied in this paper. The surface layer is oriented in one direction at 0° to provide better surface mechanical properties. The size of the samples is 190 mm × 20 mm × 2 mm. Table 1 lists the main physical performance parameters of the material.

### 2.2. Equipment

**Figure 2:**
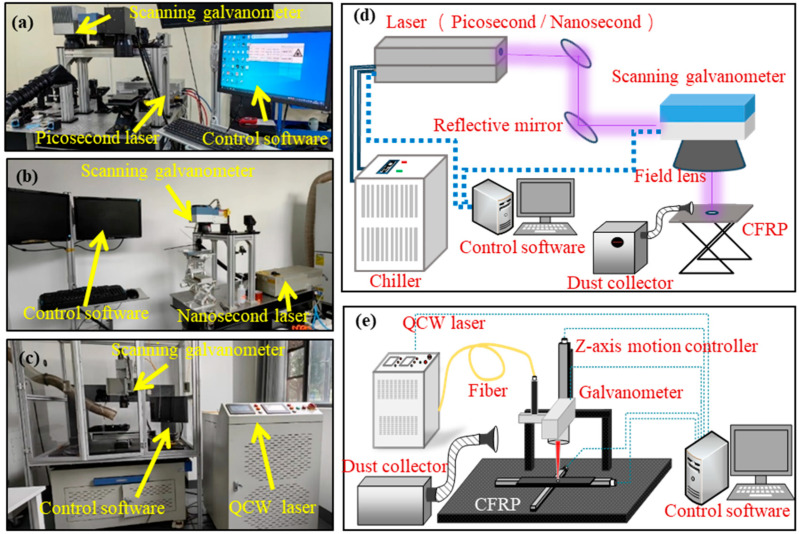
Three distinct pulse-width lasers cut system: (**a**) ultrashort-pulse laser; (**b**) short-pulse laser; (**c**) long-pulse laser; (**d**) schematic diagram of the picosecond-/nanosecond-laser system; (**e**) schematic diagram of the QCW-laser system.

To evaluate the efficiency and the quality of CFRP cutting, various pulse-width lasers were employed using the equipment shown in Figure 2. The picosecond-laser-processing platform (Figure 2a) consisted of a 355 nm picosecond ultraviolet laser (PX200-3-GHF, MKS Instruments, Andover, MA, USA), a chiller, an optical path system, a dynamic scanning galvanometer, a telecentric focusing field lens, and a control system. The nanosecond-laser-processing platform (Figure 2b) has a similar structure, but it employs a 15 W nanosecond Nd:YVO4 UV solid-state laser (Huaray Cypress 2-355-15, Wuhan, China). The QCW-laser-processing platform (Figure 2c) utilized a QCW fiber laser (YLM-450/4500-QCW-MM-AC, IPG Photonics Corporation, Marlborough, MA, USA) and laser conduction through fiber. This platform used air cooling and did not require a chiller. The scanning mirror could move vertically along the movement axis. The parameters of laser processing systems with different pulse widths are presented in Table 2.

### 2.3. Method

**Figure 3:**
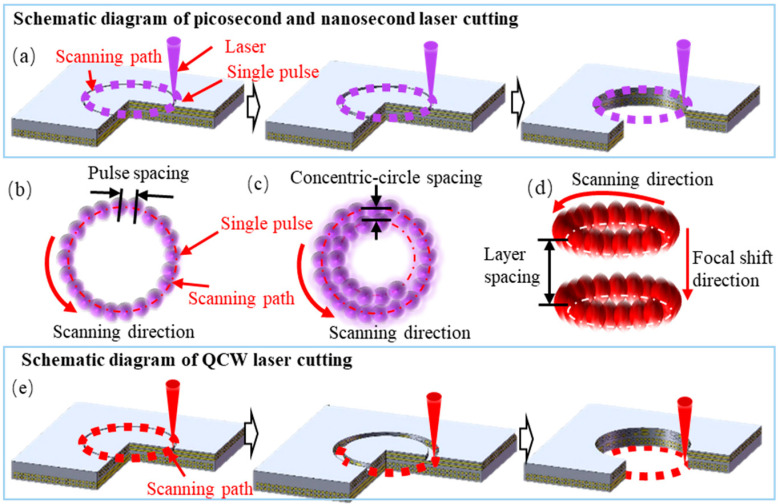
Schematic of CFRP cutting strategies with three distinct pulse-width lasers: (**a**) picosecond and nanosecond lasers; (**b**) pulse spacing illustration; (**c**) concentric-circle spacing illustration; (**d**) layer spacing illustration; (**e**) QCW laser.

As the focused laser spot diameter is much smaller than the material’s thickness, a single-pass scan cannot yield sufficient kerf width and depth. Therefore, a concentric-circle multi-pass filling strategy was adopted for laser cutting, as illustrated in Figure 3. Specifically, both the picosecond UV and nanosecond UV laser platforms operate in a fixed-focus mode: the beam scans along a series of concentric-circle trajectories with controllable spacing on the focal plane, progressively ablating the material to form an annular groove. Repeated concentric-circle passes steadily increased the ablation depth until the groove penetrated the entire thickness, causing the central plug to drop out and form a through-hole (Figure 3a). In contrast, the QCW-laser cutting process shifted the focal plane downward twice in the thickness direction to mitigate excessive surface ablation caused by repetitive scanning (Figure 3e).

The adjacent pulse spacing (Figure 3b), the spacing between adjacent concentric circles (Figure 3c), and the layer spacing of focal shifts (Figure 3d) are critical parameters governing hole quality. Excessively large spacing leaves residual matrix material, whereas excessively small spacing—that is, high pulse overlap—can induce excessive heat accumulation, resulting in carbon fiber burn-off or matrix pyrolysis. The concentric-circle spacing for each of the three laser platforms was therefore determined from preliminary experiments to ensure complete matrix removal while limiting heat-affected-zone expansion, thereby achieving high-quality laser-cut holes.

Laser power, pulse frequency, and scanning speed are key factors influencing the effectiveness of CFRP laser cutting. The picosecond laser, with its ultrashort-pulse width, significantly reduces HAZ. The quality and efficiency of hole-making are highly sensitive to variations in the scanning repetition frequency and scanning speed. The experimental parameters included scanning repetition frequencies of 400 kHz, 500 kHz, and 600 kHz, and scanning speeds of 0.3 m/s, 0.5 m/s, and 0.7 m/s. By optimizing the combination of scanning repetition frequency and speed, the pulse overlap rate can be precisely controlled, thereby effectively managing material removal efficiency and thermal accumulation. This approach enables the production of high-quality holes.

In contrast, the nanosecond laser generates significant thermal effects during processing. In addition to scanning frequency and speed, laser power also has a pronounced impact on hole quality. The experimental parameters included scanning frequencies of 60 kHz, 70 kHz, and 80 kHz, scanning speeds of 600 mm/s, 800 mm/s, and 1000 mm/s, and laser power levels of 70%, 80%, and 90% of the maximum output power. By appropriately matching power with scanning parameters, material removal rates can be increased while effectively balancing thermal damage, minimizing carbon fiber ablation, and reducing matrix resin thermal degradation. This results in high-quality hole structures.

For QCW-laser-cut, exploratory experiments revealed that too small an ablation pit leads to incomplete material separation between concentric circular trajectories, while too large an ablation pit causes excessive thermal accumulation, resulting in over-ablation of CFRP. Based on these observations, an optimal pulse width of 0.1 ms was selected. Given the longer pulse width of the QCW laser, which leads to more pronounced thermal accumulation effects, scanning frequency and laser power become the primary factors affecting laser cutting quality. The experimental parameters included scanning repetition frequencies of 600 Hz, 800 Hz, and 1000 Hz, with corresponding maximum cutting speeds of 50 mm/s, 60 mm/s, and 80 mm/s, and laser power levels of 70%, 80%, and 90% of the maximum output power. These variables were selected to conduct orthogonal experiments for picosecond-, nanosecond-, and QCW-laser cutting. The picosecond and QCW lasers each have 9 parameter combinations, while the nanosecond laser has 27 parameter combinations. The parameters and their levels are shown in Table 3. They were used to cut 6 mm diameter holes in the center of the CFRP sample, each with a unique set of process parameters.

### 2.4. Measurement

To evaluate the effectiveness and precision of three distinct pulse-width laser-cut methods, HAZ, sidewall roughness, internal thermal damage to the sidewall, and mechanical performance—specifically tensile and flexural properties—were selected as crucial indicators of processing quality. The cutting time was chosen as the key metric of efficiency.

**Figure 4:**
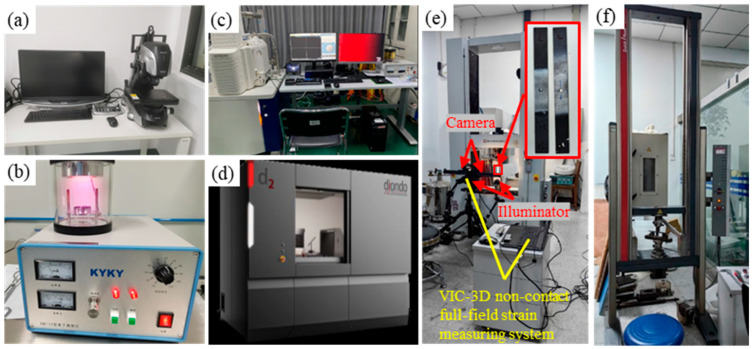
Sample inspection equipment: (**a**) optical microscope; (**b**) miniature ion sputtering device; (**c**) scanning electron microscope (SEM) and energy-dispersive X-ray spectrometer (EDX); (**d**) nano-focus industrial CT inspection system; (**e**) tensile testing machine; (**f**) flexural testing machine.

Figure 4 shows the various instruments used for measurement. The microscope (VHX-700, Keyence, Tokyo, Japan, Figure 4a) was used to observe the ablation morphology and to measure the HAZ and roughness. The HAZ was primarily characterized by measuring the width of the exposed fiber at the entrance [5], while the roughness of the inner walls was quantified using the contour measurement module of the microscope, which analyzes surface undulations by measuring the height variations along a defined path. The ion sputtering device (SBC-12, Beijing Zhongke Keyi Co., Ltd., Beijing, China, Figure 4b) was employed to coat the sample surface, thereby enhancing the imaging quality of the scanning electron microscope (SEM). The field-emission scanning electron microscope (HITACHI SU3900, Tokyo, Japan, Figure 4c), known for its high resolution, was utilized to observe the microstructure of hole walls, analyze the fracture modes of carbon fibers, and assess the removal of resin. Energy-dispersive X-ray spectroscopy (EDX) was used in conjunction with the field-emission SEM to detect elemental changes in the material and evaluate the degree of thermal degradation. Additionally, the nano-focus industrial CT inspection system (diondo d2, Shanghai Endi Detection and Control Technology Co., Ltd., Shanghai, China, Figure 4d) employed 3D reconstruction technology to detect internal defects such as delamination and porosity, and to assess hidden damage caused during the laser processing. Mechanical performance was characterized by measuring maximum tensile (AG-IC 100KN, Shimadzu Corporation, Kyoto, Japan, Figure 4e) and flexural stresses (UTM5504GD, Shenzhen SUNS Technology Stock Co., Ltd., Shenzhen, China, Figure 4f) with a material high-temperature durability testing machine and a high- and low-temperature universal creep testing machine, as shown in Figure 4. Quasi-static tensile tests were performed according to the international standard of ASTM D5766 [28] with a constant rate of 5 mm/min under normal environmental conditions. Flexural tests were conducted with a test span of 100 mm and a loading speed of 20 mm/min.

For the tensile test, a non-contact full-field strain measuring system (VIC-3D, CSI, Columbia, SC, USA, Figure 4e) was employed to study the mechanical performance and strain distribution of CFRP laminates with an open hole, including the strain of stretching direction (Y-axis) and normal to the stretching direction (X-axis), and to observe the crack growth. The 3D digital image correlation system (DIC) consisted of two CCD cameras, two light sources, a tripod stand, and a PC workstation. Uniform illumination of the specimen surface was ensured by keeping two light sources on either side of the camera. The two cameras were vertically mounted on a tripod. Cameras were then calibrated for their position and orientation using an appropriate calibration grid plate. The VIC-3D system was utilized for post-processing of recorded images to analyze full-field strain distribution. Before the DIC test, the surface of the tensile sample was painted with white paint and then speckled with black paint to obtain a high-contrast random gray distribution image, as shown in Figure 4e. A high-resolution 190 kV nanotube industrial CT detection system and 3D visualization and analysis system of CT data were utilized to observe the tensile fracture section of the sample and analyze the internal damage of the fracture section.

## 3. Results and Discussion

### 3.1. Influence of Three Distinct Pulse-Width Lasers on HAZ

**Figure 5:**
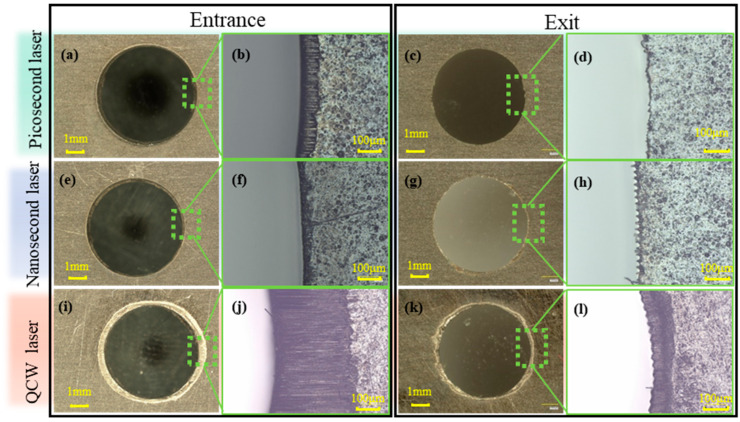
Entrance and exit morphology of holes cut by picosecond, nanosecond, and QCW lasers: (**a**–**d**) picosecond laser at 500 kHz frequency and 0.7 mm/s speed; (**e**–**h**) nanosecond laser at 80 kHz frequency, 1000 mm/s speed, and 70% power; (**i**–**l**) QCW laser at 800 Hz frequency and 70% power.

In this study, CFRP specimens were cut using picosecond, nanosecond, and QCW lasers under a multi-pass, multi-layer scanning strategy that progressively removed material layer by layer until the central plug was detached to form a through-hole. The resulting entrance and exit morphologies are shown in Figure 5. At the entrance, holes cut by picosecond lasers exhibited smooth, continuous circular edges (Figure 5b) and a narrow, band-like HAZ, where the resin was only slightly degraded and the fibers were exposed but not heavily carbonized. This minimal damage is attributed to the dominant photochemical ablation and limited thermal accumulation inherent in ultrashort pulses, although repeated scans still caused partial resin decomposition. In contrast, holes cut by a nanosecond laser displayed good entrance roundness (Figure 5f) but had an irregular HAZ at the rim with pronounced size fluctuations. These fluctuations are attributable to the moderate single-pulse energy, which partially pyrolyzed the resin and, in combination with thermo-mechanical coupling, mechanically ablated some resin, resulting in an uneven HAZ boundary. The most severe entrance HAZ was observed in holes cut by QCW lasers, which exhibited extensive resin pyrolysis and formed a distinct annular damage zone (Figure 5j). This significant damage is due to the high energy and long-pulse duration of QCW lasers, which generated substantial heat accumulation and subjected the entrance region to multiple thermal shocks, leading to severe resin degradation. At the exit, holes cut by picosecond and nanosecond lasers exhibited almost no HAZ, indicating that laser energy was efficiently absorbed during penetration and did not inflict significant thermal damage. Nevertheless, exit roundness was poor and serrated (Figure 5d,h), with serration dimensions comparable to the spot diameter, signifying insufficient energy to fully cut through the final layer, leaving trace amounts of uncut resin or carbon fiber debris on the exit rim that require post-processing; a small residual HAZ was still present at the exit of holes cut by QCW laser, but was far smaller than that at the entrance (Figure 5l), implying that the strong thermal diffusion of long-pulse lasers induced only partial resin pyrolysis at the rear surface, and the severed underlying fibers also produced pronounced serrated edges whose curvature was markedly larger than those observed at exits of holes cut by nanosecond laser.

**Figure 6:**
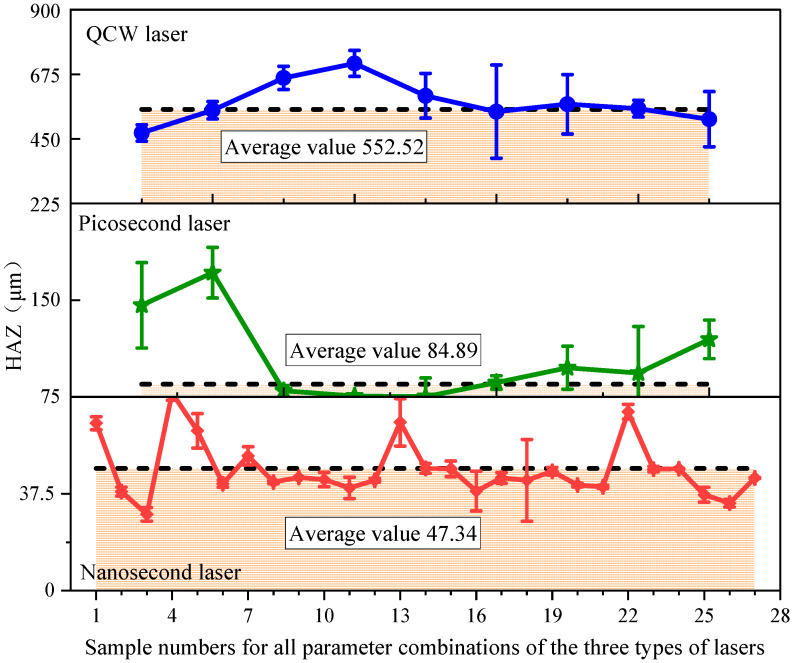
HAZ of samples cut by picosecond, nanosecond, and QCW lasers under all parameter combinations.

Figure 6 compares the HAZ values for holes cut by nanosecond, picosecond, and QCW lasers. Experimental results reveal a pronounced dependence of thermal damage on laser pulse width. Across all parameter sets, nanosecond-laser-cut specimens exhibit the smallest HAZ, followed by picosecond-laser-cut specimens, whereas QCW-laser-cut specimens yield the largest HAZ. On average, the HAZ for QCW-laser-cut holes measures 552.52 μm, which is approximately 6.5 times larger than picosecond-laser-cut holes (84.89 µm) and 11.6 times larger than nanosecond-laser-cut holes (47.34 µm). This significant difference highlights the critical role of pulse width in HAZ formation. The longer pulse duration of QCW lasers results in substantial heat accumulation, thereby expanding the affected zone. In contrast, the shorter pulses of nanosecond and picosecond lasers focus energy deposition and limit thermal diffusion, significantly reducing the HAZ. Additionally, the observed variability in HAZ values underscores the significant sensitivity to processing parameters, thereby necessitating further investigation into the interactive effects of these parameters on cutting quality for each pulse-width regime.

The HAZ ranking for the three laser cutting processes is QCW >> nanosecond > picosecond. Notably, the picosecond laser yields a slightly larger HAZ than the nanosecond laser, which contradicts common expectations. This discrepancy arises as follows: QCW-laser cutting is dominated by thermal accumulation, producing the largest HAZ; its high single-pulse energy delivers intense heat input, rapidly carbonizing the resin and pyrolyzing the fibers, while the long-pulse duration allows deep heat diffusion, and multiple scans further aggravate thermal damage, resulting in almost complete resin ablation at the entrance. Nanosecond-laser-cut, governed by coupled thermal–mechanical effects, produces a moderate HAZ; its medium single-pulse energy confines thermal impact, and the short-pulse duration limits heat diffusion, so only partial resin pyrolysis occurs. Picosecond-laser cutting possesses the lowest single-pulse energy and should theoretically induce the smallest thermal impact; however, its actual HAZ slightly exceeds that of nanosecond-laser cutting. This is mainly because the low-energy ablation depth of ultrashort pulses is shallow, requiring more scanning passes to penetrate the same thickness. Consequently, the resin at the entrance is repeatedly heated, cumulative thermal effects intensify, and successive irradiations progressively decompose the resin, forming a wider HAZ.

### 3.2. Influence of Three Distinct Pulse-Width Lasers on Sidewall Structure

**Figure 7:**
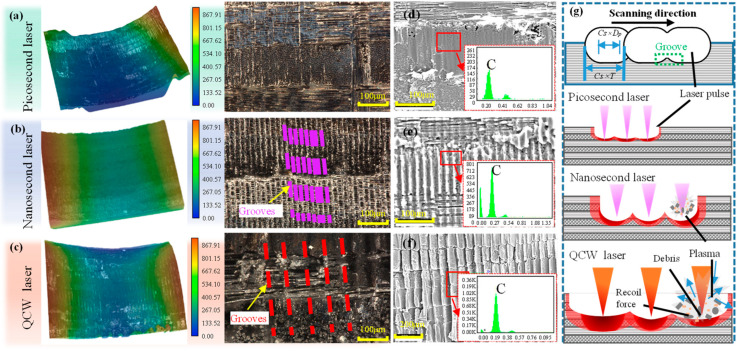
Sidewall morphology and formation diagram on holes cut by picosecond, nanosecond, and QCW lasers: (**a**,**d**) picosecond laser at 500 kHz frequency and 0.7 mm/s speed; (**b**,**e**) nanosecond laser at 80 kHz frequency, 1000 mm/s speed, and 70% power; (**c**,**f**) QCW laser at 800 Hz frequency and 70% power; (**g**) schematic of groove striation formation mechanism.

Figure 7 presents the sidewall morphologies of holes cut by nanosecond, picosecond, and QCW lasers. A regular array of grooves is clearly observed on every sidewall; these grooves correspond to the serrated protrusions along the circular edges shown in Figure 5d,h,i. The formation mechanism is governed primarily by the spot-overlap ratio during laser ablation. As the laser spot advances along the scanning direction, successive ablation craters are produced on the CFRP surface. Because only partial overlap exists between adjacent craters, material at the crater rims is not completely removed, giving rise to periodically spaced grooves on the sidewall (Figure 7g). In addition, the sidewalls of the three laser-cut specimens all appear black. The EDS analysis of the sidewalls revealed a high concentration of carbon (Figure 7d–f), indicating that the laser action on the carbon fibers led to the pyrolysis of both the carbon fibers and the resin [19,29]. This process resulted in the formation of a significant amount of carbonized residues that adhered to the sidewalls. During multi-pass annular cutting, the kerf is extremely narrow, preventing the pyrolysis products from escaping; they therefore adhere to the sidewalls and create a uniformly dark cut surface.

**Figure 8:**
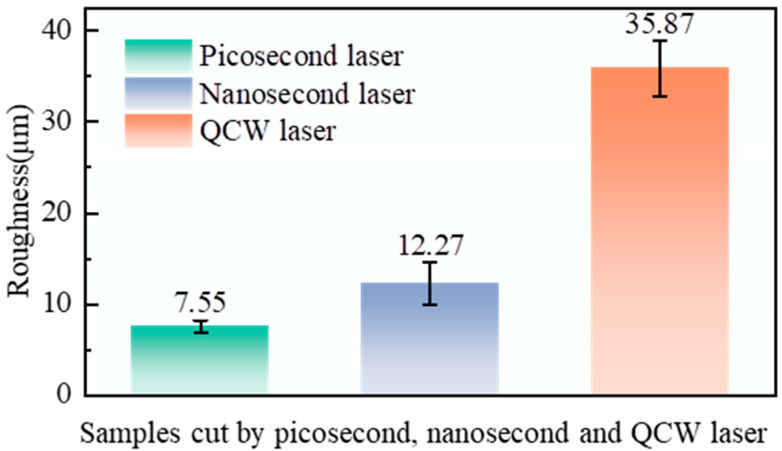
Sidewall roughness of holes cut by picosecond, nanosecond, and QCW lasers: picosecond laser at 500 kHz frequency and 0.7 mm/s speed; nanosecond laser at 80 kHz frequency, 1000 mm/s speed, and 70% power; QCW laser at 800 Hz frequency and 70% power.

The sidewall groove height of nanosecond-, picosecond-, and QCW-laser-cut holes was measured and taken as the sidewall-roughness index (Figure 8). Average sidewall-roughness values were found to be 35.87 µm for QCW-laser holes, and 7.55 µm and 12.27 µm for picosecond- and nanosecond-laser holes, respectively.

**Figure 9:**
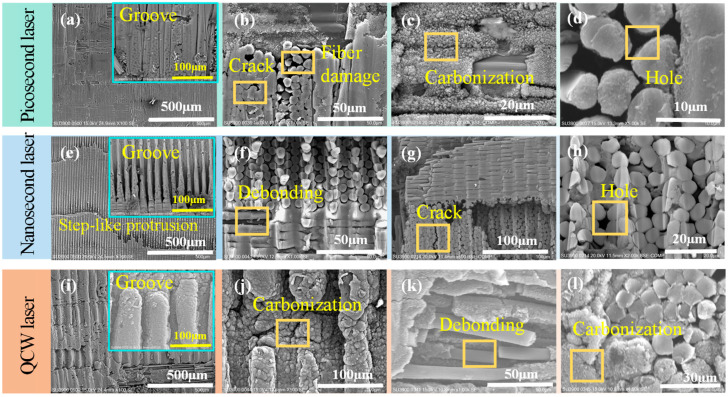
SEM micro-morphology of sidewalls on holes cut by picosecond, nanosecond, and QCW lasers: (**a**–**d**) picosecond laser at 500 kHz frequency and 0.7 mm/s speed; (**e**–**h**) nanosecond laser at 80 kHz frequency, 1000 mm/s speed, and 70% power; (**i**–**l**) QCW laser at 800 Hz frequency and 70% power.

Further systematic observation and analysis of the sidewall micro-morphologies of specimens cut by picosecond, nanosecond, and QCW lasers were conducted via field-emission scanning electron microscopy (Figure 9). High-resolution imaging distinctly reveals the disparate laser–material interaction mechanisms. SEM images in Figure 9a,e,i show that the groove width generated by successive laser pulses increases non-linearly as the pulse width extends from the picosecond through the nanosecond to the millisecond regime. Although the sidewalls cut by picosecond and QCW lasers exhibit continuous groove patterns, they differ fundamentally at the microscale: picosecond ablation is governed by ultrashort-pulse-induced non-linear absorption, yielding microscopically smooth groove edges, whereas QCW removal proceeds via thermal accumulation, leaving a pronounced carbonized layer on the groove surface. Notably, nanosecond-laser-cut specimens display distinctive step-like terraces. This morphology arises from multiple physical effects during fixed-focus deep-hole cutting: first, significant beam shadowing by the upper sidewall material, and second, intensified plasma shielding due to the longer nanosecond pulse duration, jointly producing the characteristic stepped structure. By contrast, the extremely shallow ablation depth of picosecond pulses combined with a higher number of scanning passes effectively mitigates sidewall shadowing, while QCW processing circumvents this issue through its larger ablation depth and downward focal shifting, thus maintaining continuous groove patterns. These findings not only confirm the pivotal role of pulse width in determining HAZ magnitude, but—more importantly—demonstrate that pulse-width-dependent dynamics of laser–material interactions, including plasma evolution, energy coupling efficiency, and material removal mechanisms, decisively govern the final sidewall topography.

At the 0°/90° ply interfaces of the multiscale CFRP architecture, pronounced anisotropic machining characteristics were observed for each laser regime. Picosecond-laser-cut specimens exhibited discontinuous grooves at the interface (Figure 9b) arising from direction-dependent thermal conduction: along the 0° fiber direction, a nanometer-scale carbonized layer formed by matrix pyrolysis adhered uniformly to the fibers, whereas in the 90° direction, the higher fiber volume fraction and perpendicular orientation caused preferential fiber absorption, leading to brittle fiber fracture with irregular fracture surfaces and sub-micron porous networks from intense resin pyrolysis that markedly widened the fiber spacing. The nanosecond-laser-cut specimens displayed perfectly continuous grooves across the interface (Figure 9f), indicating consistent energy deposition in both 0° and 90° plies; the clear fiber–matrix interface without carbon deposits is attributed to complete vaporization of resin and fibers by the steady nanosecond fluency, which dissipates most of the heat and minimizes conduction along the fibers. QCW-laser-cut specimens showed a transitional morphology (Figure 9j): grooves remained continuous over most regions but exhibited selective fracture at the ply interface. This behavior stems from a complex multi-physics coupling: since resin absorption at 1064 nm is 1/20 that of carbon fibers, heat is conducted primarily through the fibers; the axial (0°) thermal conductivity (~50 W/m·K) is an order of magnitude higher than the transverse (90°) value (~5 W/m·K), creating a steep pyrolysis gradient. Consequently, continuous shallow craters form in the 0° direction, whereas the 90° direction remains relatively flat. Furthermore, the entire sidewall of QCW-processed holes is uniformly coated with a pyrolytic carbon layer; magnified views (Figure 9l) reveal local spalling that exposes bare 0° fibers, while resin encapsulating these fibers has fully carbonized into a porous structure. In contrast, 90° fibers retain intact cylindrical profiles with uniform diameters, underscoring the profound influence of anisotropic thermal conduction on laser processing quality.

### 3.3. Influence of Three Distinct Pulse-Width Lasers on Internal Damage

The visible surface damage and sidewall defects of laser-cut holes were examined with optical microscopy and field-emission scanning electron microscopy. To determine whether the three pulse-width lasers induce hidden internal damage, a nano-focus industrial CT system was employed to detect subsurface defects around the holes and thus evaluate the depth of laser-induced damage.

**Figure 10:**
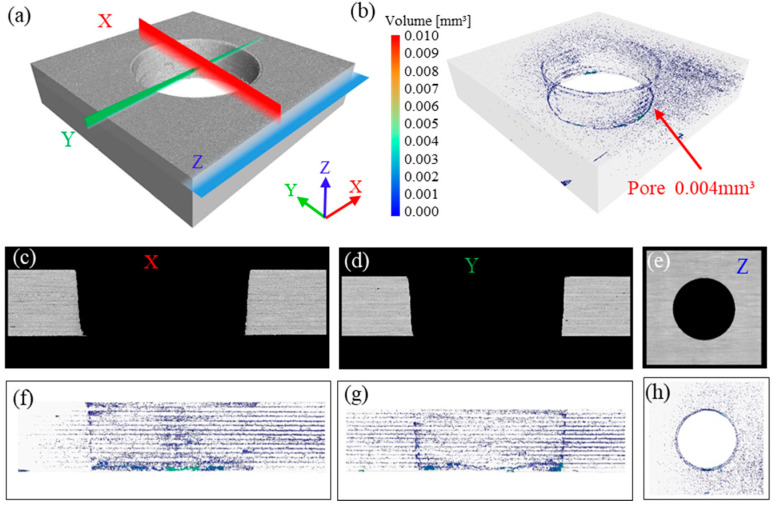
Internal-damage characterization of a picosecond-laser-cut specimen with 500 kHz frequency and 0.7 mm/s speed: (**a**) 3-D CT reconstruction of the hole; (**b**) porosity analysis around the hole perimeter; (**c**–**e**) cross-sectional view perpendicular to the X/Y/Z-axis; (**f**–**h**) pore distribution map along the X/Y/Z axis.

Figure 10 presents the CT three-dimensional reconstruction, pore quantification, and cross-sectional views along the X, Y, and Z axes for the picosecond-laser-cut specimen. The 3-D reconstruction in Figure 10a shows a smooth hole surface and sidewalls; cross-sections along the X, Y, and Z axes reveal that the structure surrounding the hole wall was identical to the interior of the CFRP plate. Figure 10b indicates that pores are concentrated at the entrance and exit HAZs and are extremely small (0.004 mm^3^), confirming that picosecond-laser cutting only causes minor resin vaporization at these locations and produces no damage within the bulk material.

**Figure 11:**
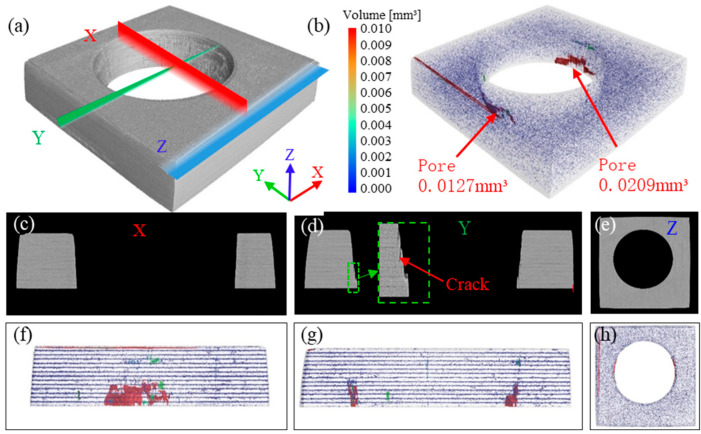
Internal-damage characterization of a nanosecond-laser-cut specimen with 80 kHz frequency, 1000 mm/s speed, and 70% power: (**a**) 3-D CT reconstruction of the hole; (**b**) porosity analysis around the hole perimeter; (**c**–**e**) cross-sectional view perpendicular to the X/Y/Z-axis; (**f**–**h**) pore distribution map along the X/Y/Z-axis.

Figure 11 shows the CT-based 3-D reconstruction, porosity assessment, and cross-sectional observations along the X, Y, and Z axes for a nanosecond-laser-cut specimen. In the 3-D reconstruction of Figure 11a, the entrance surface of the hole appears smooth, while the hole wall is uneven and undulating. Cross-sections taken perpendicular to the X, Y, and Z axes reveal that the structure around the hole wall is identical to the interior architecture of the carbon fiber laminate; in the Y-axis section, a small amount of unremoved material—i.e., the stepped protrusion noted in Figure 11e—was visible just below the hole near the exit.

Figure 11b indicates that pores are concentrated below the hole, adjacent to the exit, and are present only at the 0° azimuth. When nanosecond-laser pulses impinged on the CFRP, the material at the entrance partially shields subsequent pulses, limiting the ablation depth per pulse; consequently, some circular passes fail to fully penetrate the laminate, producing discontinuous, irregular kerfs. These kerfs correspond to the detected pores, whose volume was roughly 0.0336 mm^3^. No significant porosity was observed at the entrance or exit of the hole, demonstrating that the nanosecond-laser-cut process induces either no internal damage or only negligible damage.

**Figure 12:**
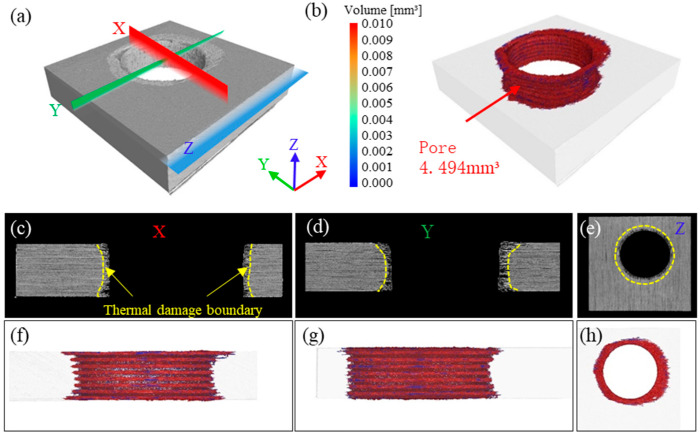
Internal-damage characterization of a QCW-laser-cut specimen with 800 Hz frequency and 70% power: (**a**) 3-D CT reconstruction of the hole; (**b**) porosity analysis around the hole perimeter; (**c**–**e**) cross-sectional view perpendicular to the X/Y/Z-axis; (**f**–**h**) pore distribution map along the X/Y/Z-axis.

Figure 12 presents the CT-based 3-D reconstruction, porosity measurement, and cross-sectional views along the X, Y, and Z axes for a QCW-laser-cut specimen. The 3-D reconstruction in Figure 12a clearly reveals an annular heat-affected zone at the hole entrance and striated grooves on the hole sidewall. In sections perpendicular to the X, Y, and Z axes, a distinct layer of thermally altered material is evident radially along the hole wall. The porosity map shows extensive pores surrounding the hole, reaching a total volume of 4.494 mm^3^; damage depth is greatest at the entrance and exit and shallowest in the middle. This indicates that heat was conducted along the fibers during QCW-laser-cut vaporization of the resin, causing pronounced internal damage to the material (Figure 12f–h).

### 3.4. Influence of Three Distinct Pulse-Width Lasers on Efficiency

**Figure 13:**
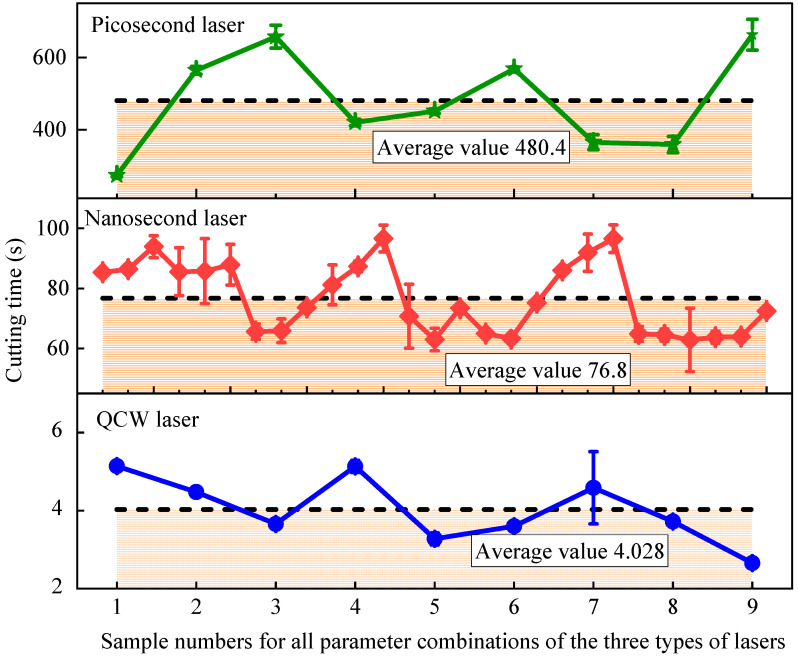
Cutting time of samples cut by picosecond, nanosecond, and QCW lasers under all parameter combinations.

To systematically evaluate the cutting efficiency disparity among picosecond, nanosecond, and QCW lasers when machining carbon fiber-reinforced composites, the present study adopts the precisely measured single-through-hole cutting time as the key performance indicator, with the statistical outcome displayed in Figure 13. Experimental data reveal an order-of-magnitude spread in processing speed: the average per-hole times decrease sharply from picosecond (480.4 s) to nanosecond (76.8 s) to QCW (4.028 s). Specifically, picosecond-laser cutting takes 119-fold longer than QCW, whereas nanosecond-laser cutting still requires 19 times the QCW duration. The physical origin of this dramatic gap lies in the three-decade difference in pulse duration—10 ps, 15 ns, and 0.1 ms—governing the transition of material removal mechanisms. Microscopic analysis shows three characteristic evolutions as pulse width lengthens: (1) the single-pulse energy-deposition zone expands, enlarging crater diameters from micrometer-scale for picosecond to hundreds of micrometers for QCW; (2) the required circumferential overlap of successive passes drops markedly, with picosecond demanding >70% overlap for acceptable quality while QCW needs only 10–40%; and (3) the focal-position tolerance rises substantially, with picosecond being exquisitely sensitive to focus shift and QCW tolerating pronounced defocus. Collectively, these effects drive an exponential increase in ablation efficiency, enabling QCW to remove material at a volumetric rate exceeding that of picosecond-laser cutting by >150× via continuous thermal accumulation. Notably, the efficiency contrast also delineates application niches: the picosecond regime, though slowest, offers cold processing and superior quality, whereas QCW’s high throughput comes with enlarged HAZs, thereby providing a pivotal efficiency-versus-quality trade-off for industrial laser-parameter optimization.

### 3.5. Influence of Three Distinct Pulse-Width Lasers on Mechanical Properties

**Figure 14:**
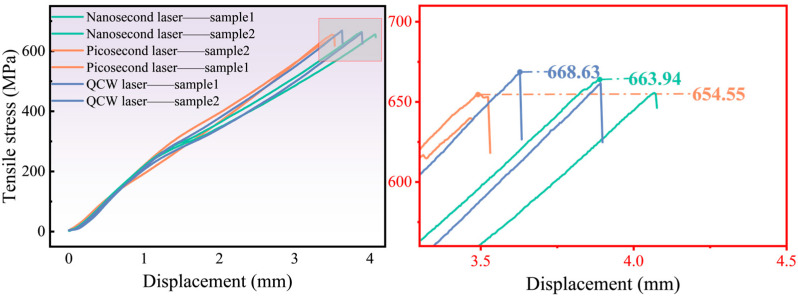
Tensile stress of specimens cut by different types of lasers: picosecond laser (600 kHz frequency and 0.5 mm/s speed); nanosecond laser (70 kHz frequency, 800 mm/s speed, and 90% power); QCW laser (1000 Hz frequency and 90% power).

Tensile stress–displacement curves for the highest- and lowest-strength specimens cut by three pulse-width lasers are presented in Figure 14. The peak tensile stresses are 663.94 MPa (picosecond), 654.55 MPa (nanosecond), and 668.63 MPa (QCW), differing by merely 2.1%. This indicates that the HAZ size around laser-cut holes exerts no appreciable influence on tensile strength. Under uniaxial loading, the tensile stress is borne almost entirely by fibers aligned with the loading direction; the resin vaporization-induced fiber exposure at the hole entrance and exit constitutes only a negligibly small fraction of the total fiber length, so the measured HAZ does not measurably degrade the tensile performance of the holed coupons.

**Figure 15:**
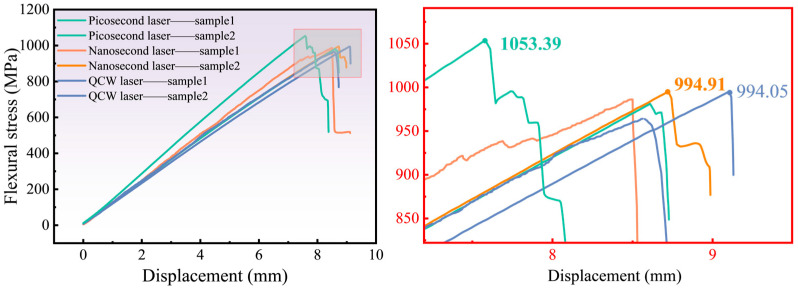
Flexural stress of specimens cut by different types of lasers: picosecond laser (600 kHz frequency and 0.5 mm/s speed); nanosecond laser (70 kHz frequency, 800 mm/s speed, and 90% power); QCW laser (1000 Hz frequency and 90% power).

Likewise, the flexural stress–displacement curves for the highest- and lowest-strength specimens cut by three pulse-width lasers are plotted in Figure 15. The peak flexural stresses are 1053.39 MPa (picosecond), 994.91 MPa (nanosecond), and 994.05 MPa (QCW). The negligible variation among these values shows that the HAZ produced by laser cutting does not appreciably degrade the material’s flexural performance. During actual bending, the upper and lower surfaces of the laminate experience compressive and tensile stresses, respectively, and this response is governed primarily by the interlaminar bond strength; none of the three laser-cut modes generated observable delamination at the interlaced fiber layers along the hole wall.

**Figure 16:**
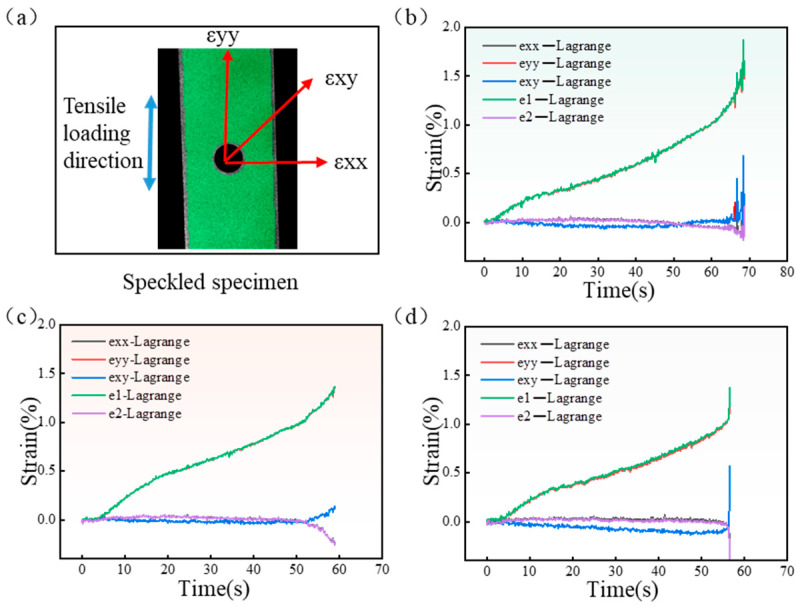
Lagrange strain curves of specimens cut by different types of lasers: (**a**) schematic diagram of the strain direction; (**b**) Lagrange strain curves of specimens cut by picosecond laser at 600 kHz frequency and 0.5 mm/s speed; (**c**) Lagrange strain curves of specimens cut by nanosecond laser at 70 kHz frequency, 800 mm/s speed, and 90% power; (**d**) Lagrange strain curves of specimens cut by QCW laser at 1000 Hz frequency and 90% power.

During the tensile loading process, the DIC system identified the full-field strain distribution and possible damage zones by speckle images before and after deformation. Figure 16 shows DIC results in terms of three in-plane strains, *ε_xx_* (transverse direction), *ε_xy_* (shear direction), *ε_yy_* (load direction), *ε*_1_ (principal strain), and *ε*_2_ (secondary strain) for CFRP laminates with an open-hole cut with various laser processing parameters. The strain curve analysis shows that *ε_yy_* is substantially consistent with *ε*_1_, and *ε_xx_* is consistent with *ε*_2_. *ε_yy_* increased monotonically with loading time and rose sharply at fracture. *ε_xx_* and *ε_xy_* do not change significantly with time, but they increase at the moment of fracture. It can be seen that *ε_yy_* of the specimen’s tensile direction is the maximum principal strain, and *ε_xx_* is the secondary strain.

**Figure 17:**
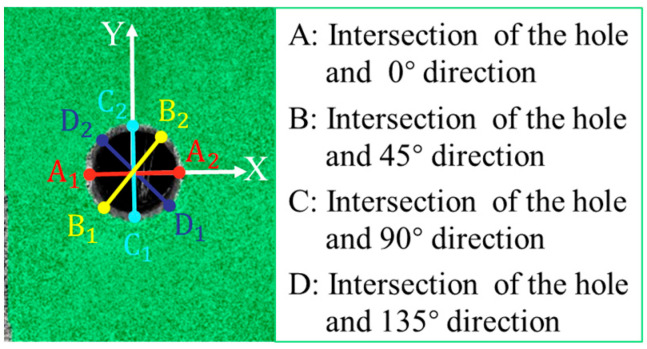
Diagram of key point setting on the laser cutting hole.

**Figure 18:**
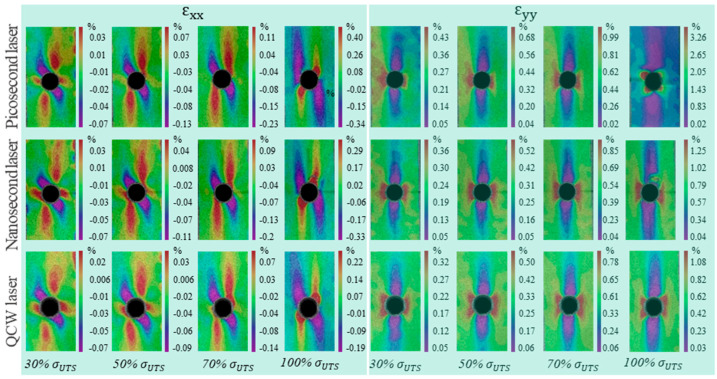
Longitudinal strain and transverse strain distribution around open holes cut by three pulse-width lasers under different tensile loads.

To facilitate the description of strain conditions, the key points at the hole edge were named, as shown in Figure 17. A, B, C, and D represent the intersection points between the directions of 0°, 45°, 90°, and 135° and the hole, respectively. Figure 18 displays the longitudinal and transverse strain distribution around the center hole of the CFRP laminate under different levels of tensile loads. Similar trends in *ε_xx_* under different loads were observed for three laser processing specimens (ps, ns, and qcw). At 30% σ_UTS_ tensile load, positive strain occurred in the region around point A and the region perpendicular to point B, and negative strain appeared in the region extending along the 135° axis at point D; the positive strain was less than the negative strain. As the tensile load increased to 50% σ_UTS_ and 70% σ_UTS_, the strain at point A decreased until it disappeared, and the positive strain near point B and the negative strain near point D gradually increased. When the tensile load increased to 100% σ_UTS_, the strain region at point B decreased sharply, and its maximum strain value was three times that of the maximum strain value at 70% σ_UTS_ tensile load. The strain region at point D expanded, and the maximum negative strain value increased slightly.

The *ε_yy_* of specimens exhibited similar strain distribution, and there was no obvious change in the strain field distribution as the tensile load increased from 30% σ_UTS_ to 100% σ_UTS_. The strain field was mainly concentrated in the vicinity of B and D, showing a hyperbolic curve shape.

**Figure 19:**
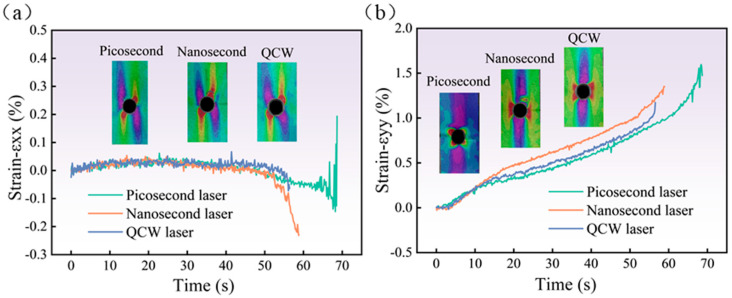
Strain diagram at point B under three distinct pulse-width laser-cut methods under 100% σ_UTS_ tensile load: (**a**) *ε_xx_*; (**b**) *ε_yy_*.

It can be observed in Figure 18 that *ε_xx_* and *ε_yy_* at point B were both significant. Point B was selected to observe the variation in *ε_xx_* and *ε_yy_* for specimens cut by the three pulse-width lasers, as shown in Figure 19. With the change in time, that is, with the increase in tensile load, *ε_xx_* at point B was not obvious, but it suddenly increased when the tensile load approached the ultimate tensile load. The variation trend in *ε_yy_* of the three laser-processing-method specimens is the same, and *ε_yy_* increased gradually with the increase in load.

**Figure 20:**
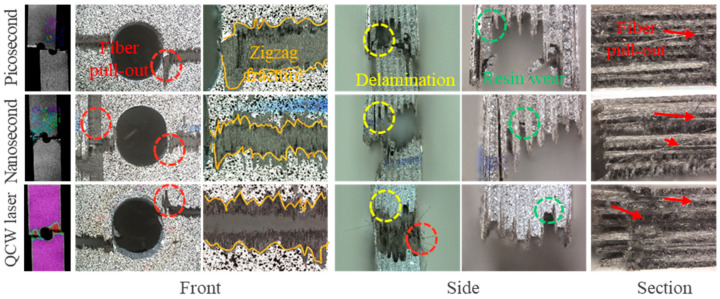
Tensile failure diagram of specimens cut by picosecond, nanosecond, and QCW lasers.

The CFRP laminate exhibited brittle fracture behavior when the applied load reached its peak level, as illustrated in Figure 20. Viewed from the front of the hole, the fracture started at the position of maximum *ε_yy_*, which was also the maximum stress concentration, and then it expanded along the X-direction. Fractures of the surface fiber (90°) presented a zigzag shape, and the fiber pull-out at the stress concentration at the hole edge is the most significant. Since the fibers had been cut by the laser, and the fiber–matrix interface strength deteriorated, the strain around the holes increased, and the stress was concentrated. The stress concentration around the hole accelerated the accumulation of damage and the propagation of cracks. The matrix cracking began at the edge of the hole, and the transverse fibers perpendicular to the load direction began to crack. With the increase in tensile load, the crack and damage area extend along the direction of the fiber, the longitudinal fiber with the same load cracks, and the final sample’s brittle fracture occurs. Upon inspecting the cross-section of tensile fracture, it can be observed that not only does the significant fiber pull-out for the stress concentration at the edge occur on the upper surface, but it is also evident in the entire thickness direction. Examination of the side fracture morphology highlights severe delamination, fiber fracture, and matrix cracking as the typical failure modes of specimen fracture. By observing the tensile fracture position from the thickness direction of the fiberboard, it is evident that the fiber layer arranged in the direction of 90° exhibits tensile fracture failure, as the tensile load of the composite porous plate structure is primarily borne by this 90° fiber layer. Conversely, the fiber layer arranged in the direction of 0° exhibits fiber–resin separation. This resin separation failure is present not only at the fracture position of the fiberboard but also within the interior of the fiber layer.

**Figure 21:**
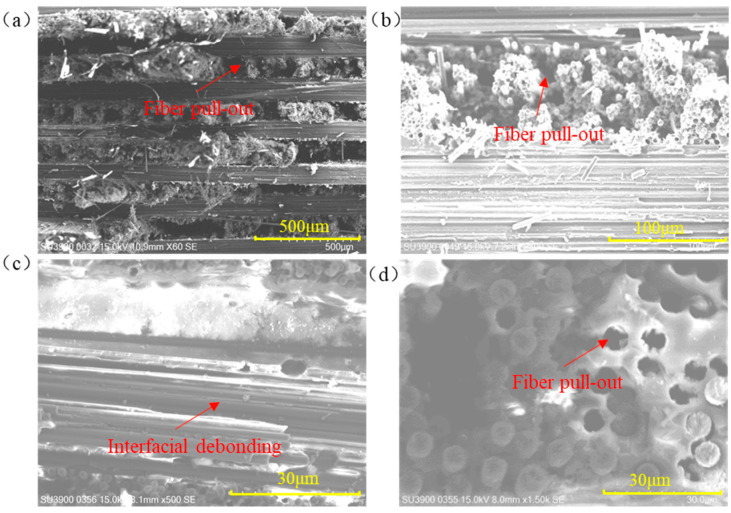
FE-SEM micrographs of the tensile-fractured interfaces: (**a**) Cross-section of carbon fiber plate fracture; (**b**) Cross-section with 0° and 90° interlaced arrangement; (**c**) Fiber layer perpendicular to the tensile direction; (**d**) Fiber layer parallel to the tensile direction.

Fracture cross-sectional images of the specimens clearly distinguish the failure modes of the fiber layers oriented parallel and perpendicular to the tensile axis. Fibers aligned with the loading direction are pulled out of the resin, producing disordered fiber bundles, whereas fibers oriented transverse to the loading direction detach from the surrounding resin and fracture under load. Multiscale characterization of the fracture surface by field-emission scanning electron microscopy (FE-SEM), as shown in Figure 21, further elucidates the microscopic failure mechanisms. Individual carbon filaments rupture under tension; because the interfacial bond between transverse fibers and the matrix was insufficient to withstand the applied load, the fibers debond along the interface, yielding relatively flat fracture surfaces (Figure 21a,c). No discernible delamination is observed between the interlaced fiber plies (Figure 21b). In contrast, fibers parallel to the loading direction exhibit fracture or pull-out, and the surrounding resin matrix develops numerous voids (Figure 21d).

### 3.6. Application Analysis of Three Distinct Pulse-Width Lasers

**Figure 22:**
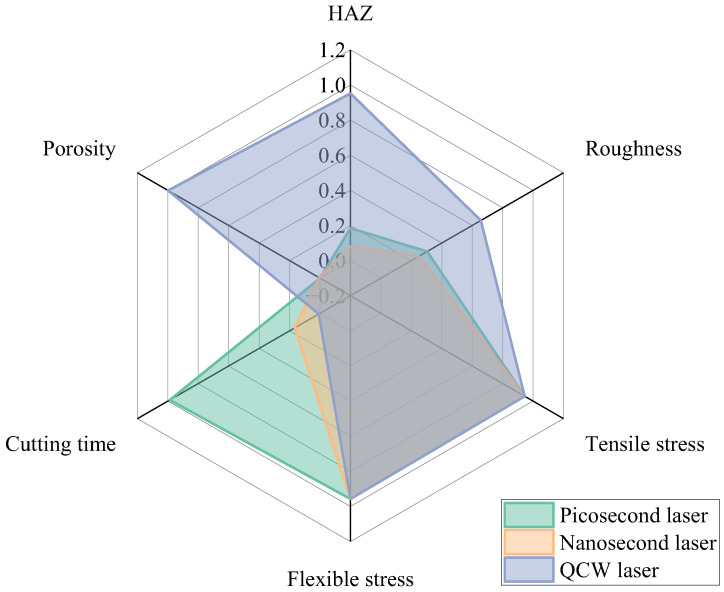
Comparison of HAZ, cutting time, sidewall roughness, flexural stress, and tensile stress for three distinct pulse-width laser cutting.

A systematic comparison of picosecond, nanosecond, and QCW lasers in CFRP hole cutting was conducted, comprehensively evaluating key metrics—including HAZ, sidewall roughness, cutting time, and flexural and tensile stress—and the consolidated results are presented in Figure 22. Experimental results reveal that the mechanical performance of specimens produced by the three laser regimes is nearly identical. Among them, nanosecond-laser-cut specimens exhibit a distinctive overall advantage: they yield the smallest HAZ width and sidewall roughness, delivering the best surface quality. In terms of processing efficiency, the average cutting time per hole cut by a nanosecond laser is only 16% of that required by a picosecond laser, demonstrating a marked efficiency gain. Consequently, nanosecond-laser machining is identified as the preferred process for precision CFRP cutting.

## 4. Conclusions

This article analyzes the efficiency and quality of CFRP hole cutting using picosecond, nanosecond, and QCW lasers. By comparing the HAZ, sidewall roughness, mechanical properties, and cutting time of specimens, the application range of picosecond, nanosecond, and QCW lasers for CFRP cutting was evaluated. The following conclusions can be drawn:

(1) The laser pulse duration decisively governs the material removal mechanism. Nanosecond pulses, with their moderate energy and duration, deliver the smallest HAZ among the three regimes; picosecond pulses, although ultrashort, suffer from limited average power and residual heat accumulation; and the QCW laser, dominated by sustained heat conduction, generates the largest HAZ (more than 11.6 times that of nanosecond pulses) and pronounced porosity.
(2) Cutting efficiency exhibits an inverse scaling with pulse width. Owing to the six-order-of-magnitude span (10 ps–0.1 ms), single-hole processing time decreases from 480.4 s (picosecond-laser cutting) to 76.8 s (nanosecond-laser cutting) and 4.028 s (QCW-laser cutting), reflecting a transition from thermally driven ablation to mechanical spallation.
(3) A multi-parameter optimization model identifies the following recommended process windows: a nanosecond laser at 200 W and 20 kHz best balances efficiency and quality (HAZ < 50 µm, cutting time < 80 s); a QCW laser is suited for coarse, rate-critical operations; and a picosecond laser remains advantageous for ultraprecision tasks.
(4) CFRP anisotropy and laser-induced damage jointly dictate failure behavior. While all laser regimes yield <5% variation in tensile and flexural strength, they markedly alter damage morphology: longitudinal plies fail by fiber pull-out, producing brush-like fracture surfaces, whereas transverse plies fail via interfacial debonding. In situ DIC and CT imaging reveal that QCW-laser-cut specimens exhibit more uniform strain fields and higher damage tolerance owing to their widened HAZ.

## Figures and Tables

**Table 1:** Thermal performance properties of CFRP plates.

Property	Units	Carbon Fiber	Epoxy Resin
Density	kg/m^3^	1800	1250
Thermal conductivity	W/(m·K)	50 (axial) 5 (radial)	0.2
Specific heat capacity	J/(kg·K)	710	1200
Vaporization temperature	K	3900	400
Volume fraction	%	67	33

**Table 2:** Parameters of laser processing systems with different pulse widths.

System Parameters	Ultrashort-Pulse Laser	Short-Pulse Laser	Long-Pulse Laser
Pulse Width (τ)	10 ps	<15 ns@60 kHz	0.05~50 ms
Wavelength (λ)	355	355 nm	1070
Beam Quality (M^2^)	≤1.2	≤1.2	5 mm × mrad
Spot Diameter (d_0_)	6.8 μm	37 μm	74 μm
Repetition Rate (F)	~1 MHz	10~200 kHz	0~50 kHz
Average Power (P_a_)	35 W	>15 W@60 kHz	450 W
Peak Power (P_p_)	-	-	4500 W

**Table 3:** Experimental design for CFRP cutting using three distinct pulse-width lasers.

Cutting Methods	Parameter 1		Parameter 2		Parameter 3	
Picosecond	Frequency (kHz)	400, 500, 600	Speed (m/s)	0.3, 0.5, 0.7		
Nanosecond	Frequency (kHz)	60, 70, 80	Speed (mm/s)	0.3, 0.5, 0.7	Power (%)	70, 80, 90
QCW	Frequency (Hz)	600, 800, 1000	Power (%)	70, 80, 90		

## Data Availability

The original contributions presented in this study are included in the article. Further inquiries can be directed to the corresponding author.
